# Multimodal evidence for bone lymphatics in skeletal health and repair

**DOI:** 10.1016/j.cell.2026.06.026

**Published:** 2026-08-20

**Authors:** Yang Yang, Yuheng Zhang, Yixin Shi, Sanyam Jain, Jessy D. Joseph, Amit Singh, Junyu Chen, Saravana K. Ramasamy, Anjali P. Kusumbe

**Affiliations:** 1 Tissue and Tumor Microenvironments Laboratory, Cancer Discovery and Regenerative Medicine Program, Lee Kong Chian School of Medicine, Nanyang Technological University, Singapore 636921, Singapore; 2 Multidisciplinary Institute of Ageing, University of Coimbra, 3005-504 Coimbra, Portugal; 3 State Key Laboratory of Oral Diseases, National Center for Stomatology, National Clinical Research Center for Oral Diseases, West China Hospital of Stomatology, Sichuan University, Chengdu 610041, Sichuan, China; 4 Nutrition, Metabolism and Health Research Program, Lee Kong Chian School of Medicine, Nanyang Technological University, Singapore 636921, Singapore

**Keywords:** bone lymphatics, lymphatic endothelial cells, skeletal vasculature, skull angiogenesis, aging, bone vasculature, bone regeneration, single-cell RNA sequencing, spatial transcriptomics, light-sheet imaging

## Abstract

Previous studies predominantly associated lymphatics with skeletal disease and bone loss. However, building on our work, bone lymphatics are emerging as a paradigm-shifting component of the skeletal microenvironment, illustrating their role as positive regulators of bone mass and repair. Here, we present a comprehensive analysis integrating spatial transcriptomics, single-cell RNA sequencing, and imaging across murine and human bones. Spatial transcriptomics identifies Prox1^+^ endothelial cells embedded within bone. Reanalysis of multiple scRNA-seq datasets confirms Prox1^+^ lymphatic endothelial cells (LECs) in bones, despite their underrepresentation in soft-tissue endothelial cell atlases. Periosteum is an insufficient source for bone lymphatics because it contains only sparse LECs. Our analyses further demonstrate that certain mouse models lack the sensitivity required to detect bone lymphatics and highlight the importance of high-resolution imaging. Collectively, convergent multimodal evidence substantiates LECs as an integral functional component of the skeletal microenvironment. This Matters Arising Response paper addresses the Meng et al. (2026) Matters Arising paper, published concurrently in this issue.

## Highlights

• Certain mouse models miss bone lymphatics
• Multimodal analyses support bone lymphatics
• Periosteum is an insufficient source
• Converging evidence establishes that bone lymphatics regulate regeneration and bone mass

## Graphical abstract

**Figure:**
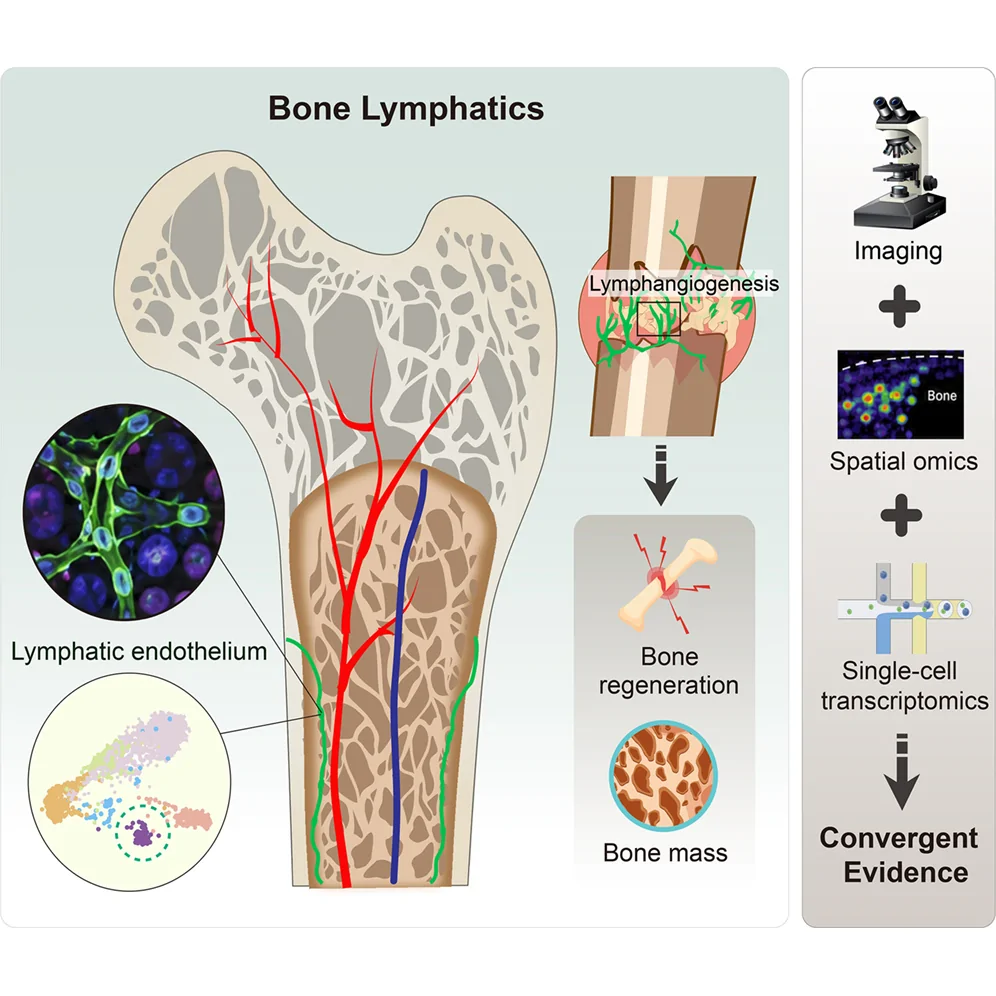

## Teaser

Spatial transcriptomics, single-cell transcriptomics, and high-resolution imaging support bone lymphatics as integral components of the skeletal microenvironment and provide a framework for resolving their identity and detection. This paper is a response to the Matters Arising article by Meng et al. in this issue of *Cell*.

## Introduction

The existence of lymphatic vessels within skeletal tissues has long been debated due to persistent technical challenges in bone research, including limited antibody penetration, optical scattering from the mineralized matrix, and restricted imaging depth. We provided direct evidence of lymphatic vessels embedded within both cortical bone and the marrow compartment, using advanced genetic mouse models and light-sheet fluorescence microscopy.^1^

Since this initial demonstration, multiple independent studies using complementary approaches have substantiated and extended the presence and functional relevance of bone lymphatics.^2^^,^^3^^,^^4^^,^^5^^,^^6^^,^^7^^,^^8^^,^^9^^,^^10^ Lymphatic vessels and their involvement in age-associated osteolysis have been shown, while single-cell transcriptomic analyses have identified lymphatic endothelial cells (LECs) within bone.^3^^,^^11^^,^^12^^,^^13^ Lymphatic vessels limit inflammatory bone loss.^14^^,^^15^ VEGF-C/VEGFR3 (Vascular Endothelial Growth Factor C and Vascular Endothelial Growth Factor Receptor 3) signaling limits osteoclast activation in periprosthetic osteolysis, but aging impairs this via SASP (Senesence-Associated Secretory Phenotype) from senescent mesenchymal stem cells (MSCs)—effects reversible with JAK (Janus Kinase) inhibitors. LEC apoptosis worsens bisphosphonate-related jaw osteonecrosis, whereas parathyroid hormone (PTH) enhances lymphangiogenesis and bone formation. Under pathological conditions, including bisphosphonate-related osteonecrosis of the jaw, marked rarefaction of lymphatic vessels leading to disease progression has been observed, showing jaw-specific evidence for lymphatic involvement.^10^^,^^16^^,^^17^ A growing body of work in bone repair and bone regeneration demonstrates that lymphangiogenesis promotes coordinated angiogenesis, immune remodeling, and bone formation.^2^^,^^3^^,^^18^^,^^19^^,^^20^^,^^21^^,^^22^^,^^23^ Increased VEGF-C signaling enhances bone formation and improves bone quality, whereas impaired lymphatic drainage and reduced lymphangiogenesis compromise fracture repair.^2^^,^^24^^,^^25^ Conversely, enhanced lymphatic function and lymphangiogenesis support reparative immune responses, osteoblast survival, and bone formation. Collectively, these independent studies establish lymphatic vessels and lymphangiogenesis as a reproducible and functionally relevant component of the bone microenvironment in bone repair and aging and disease-related dysfunction. Notably, all these studies stand in clear contrast to studies from authors proposing that lymphangiogenesis drives bone loss.^26^^,^^27^

The concerns raised regarding the mouse lines should be considered in the broader context of approaches used across other studies. Similarly, hematopoietic stem cells, which are conventionally identified by a combination of markers, were identified mainly using only CD150, and this CD150 immunostaining lacked cell-surface localization.

To further understand these discrepancies, we pragmatically evaluated their concerns and the data presented. We employed a comprehensive, multimodal analysis integrating light-sheet imaging and both single-cell and spatial transcriptomics across a wide array of murine and human skeletal tissues (Figure S1). Our findings provide robust, convergent evidence that substantiates lymphatic vasculature as an intrinsic and functionally significant component of the bone microenvironment.

## Results

### Spatial transcriptomics confirms Prox1-expressing ECs in bone

In our study, we demonstrated that LECs expand during bone injury and actively contribute to skeletal repair and regeneration. By contrast, Meng et al. report that Prospero Homeobox 1 (*Prox1*)^+^ endothelial cells (ECs) are not detected in either homeostatic or injured bone.^28^ Spatial transcriptomics offers a high-resolution approach for mapping transcriptionally defined cell populations within intact tissue architecture. To independently evaluate the presence and spatial distribution of Prox1 expression and *Prox1*^+^ LECs in bone, we analyzed two orthogonal spatial transcriptomics datasets derived from validated mouse models of bone injury and regeneration (Methods S1).^29^^,^^30^

**Figure 1:**
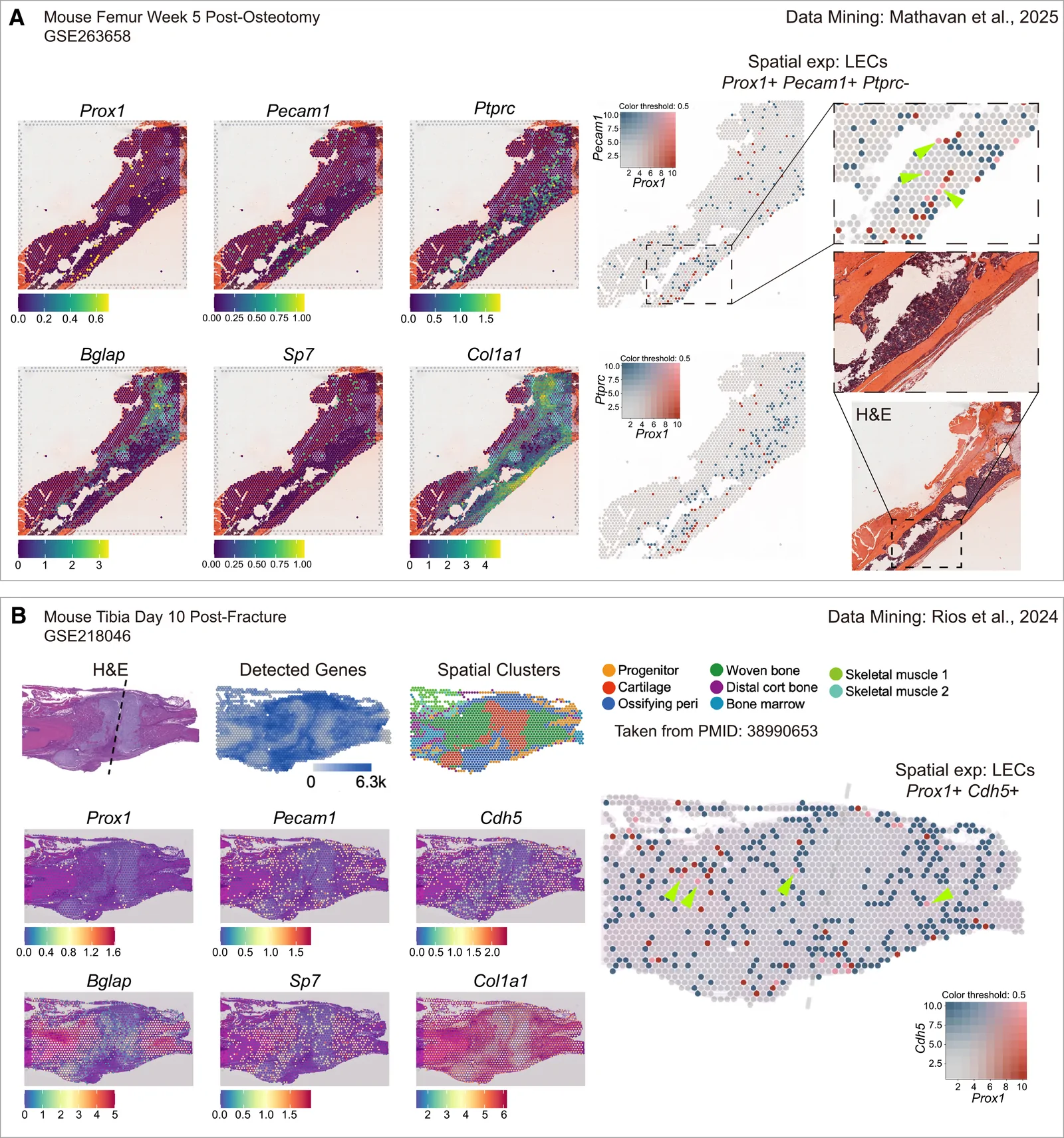
Spatial transcriptomics confirms the presence of Prox1^*+*^ ECs in bone (A) Spatial feature plots show the expression of *Prox1*, *Pecam1*, *Ptprc*, *Bglap*, *Sp7*, and *Col1a1*. Co-expression of *Prox1* and *Pecam1*, while these cells lack *Ptprc* expression. Green arrowheads indicate *Prox1*^+^*Pecam1*^+^*Ptprc*^*−*^ LECs in the cortical bone and marrow. (B) Spatial transcriptomics data with a dashed line indicates the fracture plane. Spatial feature plots display *Prox1*, *Pecam1*, *Cdh5*, *Bglap*, *Sp7*, and *Col1a1* expression. Double-positive *Prox1*^+^*Cdh5*^+^ LECs are shown and indicated by green arrowheads. Adapted from Zhang et al., *J Adv Res*, 2026, with permission from Elsevier (license no. 6310651104895).

In the dataset from Mathavan et al., spatial transcriptomics was performed on femoral sections following bridged osteotomy.^29^ *Prox1*^+^ expression was detected within both the cortical bone and bone marrow compartments. Spatial feature plots revealed co-localization of *Prox1* with *Pecam1* and an absence of *Ptprc* expression in these *Prox1* and *Pecam1* double-positive cells. These *Prox1*^+^ *Pecam1*^+^ *Ptprc*^*−*^ LECs were localized in cortical bone and bone marrow (Figure 1A). By contrast, Meng et al. did not detect any *Prox1*^+^ expression in bones. These LEC spots observed in spatial transcriptomics were also positive for other lymphatic markers such as *Flt4*, *ReIn*, and *Ptgs1*. The LECs are located within bone and bone marrow, which was confirmed by the presence of neighboring *Ptprc*^+^ hematopoietic cells and bone cells, as detected by the markers *Runx2* and *Bglap* (Figure S2).

Similarly, in the dataset from Rios et al., spatial transcriptomic profiling was performed on day 10 post fracture/injury.^30^ Visualization of the spatial expression of *Prox1* showed localization of *Prox1* expression within the fracture callus and the regenerating woven bones. Spatial gene expression overlays revealed distinct localization of endothelial markers (*Pecam1* and *Cdh5*), the lymphatic marker *Prox1*, and osteolineage markers (*Bglap*, *Sp7*, and *Col1a1*). Notably, *Prox1*^+^ *Cdh5*^+^ LECs were clearly localized within the regenerating woven bone, providing direct spatial and molecular evidence for LECs in the injured bone microenvironment (Figures 1B and S3A–S3D). The LEC spots identified by spatial transcriptomics were also positive for additional lymphatic markers, including *Flt4*, *Ptgs1*, and *Sox18*. The localization of LECs within bone was further confirmed by the presence of neighboring bone cells, identified by the expression of *Runx2* and *Bglap* (Figures S3A–S3D). Consistent with these observations, Rios et al. reported these regions to correspond to newly woven bone (Figures S3A–S3D). Together, these two independent spatial transcriptomics datasets offer robust, high-resolution validation of Prox1 expression within the cortical bone and bone marrow compartments.

### Assessment of lymphatic detection and recombination efficiency in genetic mouse models

Mouse lines used in Meng et al. were previously used in Han et al. and subsequently employed in Liu et al.^31^^,^^32^ While genetic tools offer high specificity, incomplete recombination and tissue variability can still pose a challenge. Previous work highlights Dre’s slower recombination kinetics, necessitating extended analysis timelines.^33^^,^^34^^,^^35^^,^^36^ In Han et al.,^31^ embryonic tissues were analyzed 4 days post-induction, compared with 1–2 days of induction required for Cre in the embryo. Authors in their previous study where they have employed this mouse model, Liu et al.,^32^ examined adult tissues 6–12 weeks after tamoxifen induction. By contrast, Meng et al.’s analysis of bone tissues in adult mice was performed 5 days post-induction.^37^ Moreover, Meng et al. do not detect lymphatic vessel networks in well-documented regions with high lymphatic vessel density, such as joints and muscles^37^^,^^38^^,^^39^^,^^40^^,^^41^ (Figure S4). Extensive literature confirms dense lymphatic vessel networks in knee joints,^42^^,^^43^^,^^44^^,^^45^^,^^46^^,^^47^ which are less accessible than muscles and connective tissues, suggesting a fundamental methodological limitation in their tissue analysis. Moreover, tdTomato’s exceptional brightness (extinction coefficient: 138,000 M^−^¹ cm^−^¹; quantum yield: 0.69) and long-wavelength emission (581 nm) enable direct fluorescence imaging with minimal autofluorescence, eliminating the need for antibody-based detection.^48^ The reliance on immunostaining to detect tdTomato across tissues may reflect limitations related to the reporter line and/or tissue processing, which could in turn affect the ability to resolve fine structural details.

### Extended induction does not improve recombination efficiency in genetic mouse models

The experimental strategy extends the analysis to 1 month after tamoxifen injection. However, the newly provided data further show that even after 1 month of induction, the muscle displays only sparse lymphatic labeling. Similar sparse lymphatics are also detected within the bones in light-sheet imaging data (Figure S4). This observation contrasts with prior literature demonstrating a dense and continuous lymphatic network in muscle, readily detectable.^37^^,^^38^^,^^39^^,^^40^^,^^41^ Given that muscle is highly accessible and consistently exhibits dense lymphatic labeling, this is consistent with inefficient recombination in the mouse models used. Joint regions are cropped in the images presented for after 1 month of tamoxifen, precluding any assessment of lymphatics. Throughout the study, only a limited number of *Prox1*^+^ cells were observed within the joint region. Notably, these *Prox1*^+^ cells do not exhibit clear or continuous vascular structures, which may be consistent with incomplete recombination. In addition, these cells lack LYVE1 (Lymphatic Vessel Endothelial Hyaluronan Receptor 1) expression, and no evidence is provided to establish a bona fide lymphatic endothelial identity. Similar sparse lymphatics are also detected within the bones in light-sheet images (Figure S4). Taken together, the experimental approach does not appear to address the underlying limitations of the mouse model. Instead, the data are consistent with inefficient recombination in both muscle and joint tissues (Figure S4).

### scRNA-seq datasets confirm lymphatic endothelium in murine bone and bone marrow

Meng et al. reanalyzed our single-cell RNA sequencing (scRNA-seq) dataset and reported that LECs are absent in bones.^28^ To rigorously assess this, we systematically re-examined multiple independent publicly available scRNA-seq datasets (Methods S1) and consistently identified *Prox1*^+^ LECs in both the bone and bone marrow compartments. ECs in all our analyses were defined as *Cdh5*^+^, *Pecam1*^+^, and CD45 (*Ptprc*) negative. We restricted LECs as the *Prox1*-high (*Prox1*^*hi*^) cells, defined as cells with expression levels exceeding the mean by 2 standard deviations (SD) of total ECs.

**Figure 2:**
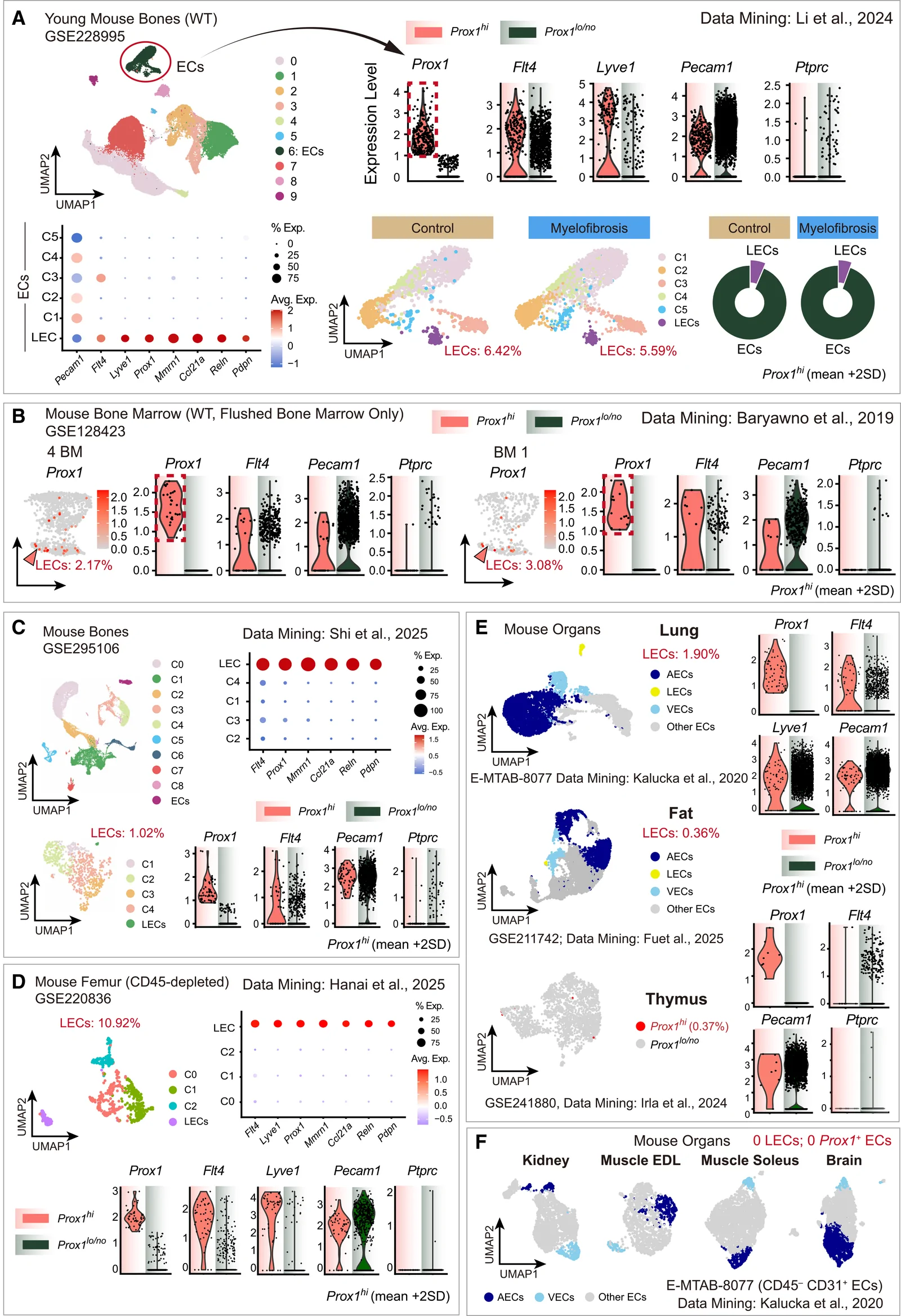
Multiple scRNA-seq datasets confirm LECs in mouse bone and bone marrow (A) Violin plots present the expression of *Prox1*, *Flt4*, *Lyve1*, *Pecam1*, and *Ptprc* in *Prox1*^hi^ and *Prox1*^lo/no^ ECs. (B) Feature plots show the expression of *Prox1* within ECs, with *Prox1*^+^ LECs marked. Violin plots present *Prox1*, *Flt4*, *Pecam1*, and *Ptprc* in *Prox1*^hi^ and *Prox1*^lo/no^ ECs. LECs were defined as *Cdh5*^+^*Pecam1*^+^*Ptprc*^*−*^*Flt4*^+^*Prox1*^+^. (C) LECs were defined as *Cdh5*^*+*^*Pecam1*^*+*^*Ptprc*^*−*^*Flt4*^*+*^*Prox1*^*+*^. Dot plot shows the expression of marker genes across different EC subsets. Violin plots present the expression of *Prox1*, *Flt4*, *Pecam1*, and *Ptprc* in *Prox1*^hi^ and *Prox1*^lo/no^ ECs. (D) Dot plot shows the expression of marker genes across different EC subsets. Violin plots present the expression of *Prox1*, *Flt4*, *Lyve1*, *Pecam1*, and *Ptprc* in *Prox1*^hi^ and *Prox1*^lo/no^ ECs. (E) scRNA-seq datasets from soft tissues. Violin plots present the expression of *Prox1*, *Flt4*, *Lyve1*, and *Pecam1* in *Prox1*^hi^ and *Prox1*^lo/no^ ECs. (F) scRNA-seq data profiling CD45^*−*^ CD31^+^ ECs from multiple mouse organs. UMAP plots show the distribution of EC subsets.

In the dataset from Li et al., which profiled non-hematopoietic stromal populations from young wild-type mice, unsupervised clustering revealed a distinct population of ECs.^49^ Within this cluster, a subset co-expressed canonical lymphatic markers, *Prox1*, *Flt4*, and *Lyve1*, alongside pan-endothelial genes *Cdh5* and *Pecam1*, while lacking the hematopoietic marker *Ptprc* (Figures 2A and S3E). This transcriptional profile is consistent with a definitive lymphatic endothelial identity. Importantly, these *Prox1*^*hi*^ LECs were detected in both control and myelofibrotic bones. A comparison of control versus mice with myelofibrosis showed a decline in LECs during myelofibrosis. Furthermore, bone cell preparations lacked expression of myogenic markers.

We also reanalyzed the stromal dataset from Baryawno et al., in which bone marrow cells were isolated by flushing long bones, a method that avoids digesting cortical bone, analyzing cells present only within the marrow cavity.^50^ Despite the exclusion of bone-embedded stromal elements, we detected *Prox1*^*hi*^ LECs in both pooled and individual samples (Figure 2B). This finding shows that LECs are resident within the marrow compartment itself and are not simply remnants of peripheral lymphatic structures or artifacts of bone digestion protocols.

We next analyzed murine mandible datasets^10^ and femoral datasets from Hanai et al. generated with depletion of CD45^+^ cells (Figures 2C and 2D). Unbiased clustering of the scRNA-seq data delineated diverse stromal and immune populations, with ECs clearly resolved based on canonical expression of *Cdh5*. Subsequent sub-clustering of ECs revealed a transcriptionally distinct subpopulation corresponding to LEC identity. LECs were stringently defined by the co-expression of *Cdh5*, *Pecam1*, *Flt4*, and high levels of *Prox1* (*Prox1*^*hi*^) (Figures 2C and 2D).

### Soft-tissue endothelial atlases reveal general limitations in LEC detection

**Figure 3:**
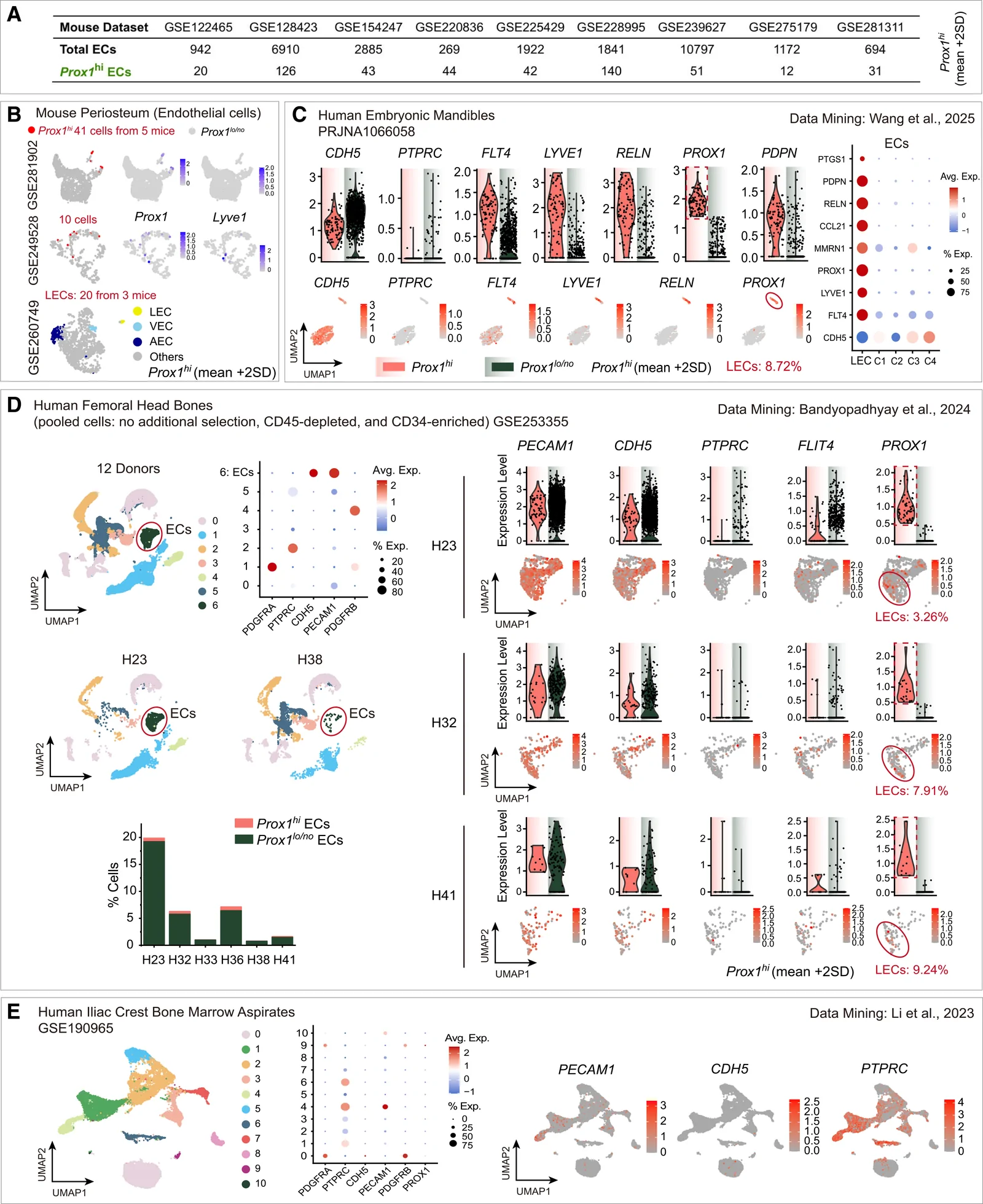
scRNA-seq datasets show LECs in bone and bone marrow (A) Table showing *Prox1*^hi^ ECs across mouse datasets. (B) Analysis of LECs in publicly available scRNA-seq datasets of periosteum. (C and D) scRNA-seq data generated from human embryonic mandibles and adult femurs. Violin plots show the expression of *CDH5*, *PTPRC*, *FLT4*, *LYVE1*, *RELN*, *PROX1*, and *PDPN* in *Prox1*^hi^ and *Prox1*^lo/no^ ECs. (E) scRNA-seq data profiling human iliac crest bone marrow aspirates.

Analysis of publicly available soft-tissue EC atlases, derived from tissues with well-established lymphatic networks, such as skeletal muscle, kidney, thymus, lung, and brain, reveals a strikingly similar or even more pronounced challenge in capturing LECs. Despite targeted enrichment of ECs in these datasets, *Prox1*-expressing cells frequently remain sparse, fail to resolve into a distinct transcriptionally coherent LEC cluster, or are markedly underrepresented (Figures 2E and 2F) in tissues such as skeletal muscle, kidney, and brain. This has important implications for the periosteal or surrounding muscle tissue contamination hypothesis, which posits that LECs detected in whole-bone preparations arise from inadvertent inclusion of surrounding soft tissues. However, this is not supported by the data: bone-derived datasets consistently show *Prox1*^*hi*^ LECs (Figure 3A), whereas carefully curated soft-tissue endothelial atlases, including that of skeletal muscle, fail to robustly capture comparable populations. Instead, the opposite pattern is observed. These findings indicate that technical constraints in LEC detection, including dissociation biases, low abundance, and limited sensitivity of current scRNA-seq pipelines, are broadly shared across tissues. Collectively, these results support the interpretation that *Prox1*^*hi*^ cells identified in bone represent bona fide bone- and marrow-resident LECs.

### Periosteum is an insufficient source of Prox1^+^ LECs and cannot account for bone-associated lymphatic endothelial populations

Analysis of publicly available periosteal tissues’ single-cell sequencing datasets further argues against the notion that periosteal contamination accounts for the LEC population observed in bone datasets. Periosteal stromal cells contain only sparse *Prox1*^+^ cells (Figure 3B). Thus, these *Prox1*-expressing cells in the periosteum are quantitatively insufficient to explain the reproducible population of *Prox1*^*hi*^ cells detected in bone and bone marrow datasets. Moreover, periosteal datasets contained *Prox1*^hi^ cells lacking detectable *Lyve1* expression (Figure 3B). Importantly, the frequency of *Prox1*-expressing cells in periosteum is far too low to account for the abundance and consistent detection of *Prox1*^*hi*^ LECs identified within bone and marrow compartments across datasets. Moreover, periosteal *Prox1*^+^ cells do not resolve into a distinct lymphatic endothelial cluster in all single-cell atlases (Figure 3B), further supporting their limited biological contribution. Together, these observations indicate that the periosteum cannot serve as a substantive or primary source of the *Prox1*^*hi*^ lymphatic endothelial population detected in bone single-cell sequencing datasets.

### scRNA-seq confirms lymphatic endothelium in human bone and bone marrow

Next, we analyzed the dataset from Wang et al.^18^^,^^51^ that profiled human embryonic mandibles.^51^ In our analysis for bone LECs, we restricted LECs as the *PROX1*-high (*PROX1*^*hi*^) cells, defined as cells with expression levels exceeding the mean by 2 SD of total ECs. Within endothelial clusters, we observed robust co-expression of canonical LEC markers, including *PROX1*, *LYVE1*, *CDH5*, and *FLT4*. Feature and violin plots demonstrated marker co-localization in discrete EC subsets, confirming their lymphatic endothelial identity (Figure 3C). Together, the integration of mouse and human datasets derived from multiple independent sources, diverse scRNA-seq platforms, and varied tissue processing workflows, including embryonic and adult tissues, provides compelling evidence that *Prox1*^*hi*^ LECs are a consistent and bona fide component of the bone and bone marrow microenvironment.

We next assessed human bones for the presence of lymphatic vessels. In the femoral head, a weight-bearing long bone, analysis of the Bandyopadhyay et al. dataset revealed *PROX1*^+^ *CDH5*^+^ *PECAM1*^+^ *PTPRC*^*−*^ ECs across multiple donor samples.^52^ *FLT4* expression was not consistently detected in both ECs and LECs; however, the *PROX1*^*hi*^ *CDH5*^+^ cell cluster was reliably present within the EC compartment, supporting the existence of LECs in human bones (Figure 3D). These 12 human samples exhibited variability in EC abundance, with LECs being very low in samples with very low EC representation (Figure 3D). As human samples are not whole-bone and whole-marrow samples, our analysis across multiple datasets showed high variability. Moreover, there are datasets that recovered a low fraction of *CDH5*^+^ EC fraction, suggesting potential region-specific differences (Figure 3E).

Collectively, the public human datasets spanning embryonic and adult skeletal sites provide compelling evidence that *PROX1*^+^ LECs are a consistent component of the human bone microenvironment.

### Field-wide methodological considerations in identifying bone lymphatic endothelium

Meng et al. note that genetic ablation of LECs using *Prox1-CreERT2*-driven DTA may have limitations. However, PROX1 is the master regulator of lymphatic endothelial identity. Thus, *Prox1-CreERT2*-driven DTA ablation provides a functionally relevant strategy to target bona fide lymphatic lineage cells. Importantly, this approach enables causal interrogation of lymphatic function in adult bones, which is not achievable through marker-based analyses alone. They also raise concerns that restricted marker sets may be insufficient to define endothelial populations in bone. In prior studies, endothelial sub-populations were typically identified using two to three broadly expressed markers and, in some cases, only two markers. In our study, lymphatic vessels were identified through high-resolution three-dimensional light-sheet imaging combined with co-expression of multiple established lymphatic endothelial markers (LYVE1, PDPN, and PROX1) across whole bones. While no single marker is entirely exclusive to lymphatic endothelium, this limitation reflects a general constraint of marker-based identification rather than a study-specific issue. Importantly, our approach integrates marker co-expression with 3D vessel morphology, spatial continuity, and whole-bone context, providing enhanced anatomical and contextual resolution compared with standard confocal microscopy.^53^ Whole-bone preparations for scRNA-seq and proteomic profiling are a standard and widely adopted approach in bone and bone vascular biology. This strategy has been extensively used to define stromal, mesenchymal, and endothelial heterogeneity. Therefore, similar considerations regarding potential periosteal inclusion apply broadly across published bone and bone vascular datasets. This reflects a widely accepted methodological framework in the field.

### Pathology-associated lineage tracing limits inference of physiological lymphatics

While lymphatic vessels are difficult to detect in histological sections of healthy bone, they become more readily observable under pathological conditions, where they not only expand but also upregulate expression of lymphatic markers such as *Lyve1* and *Prox1*, as also observed in datasets from Meng et al.^28^

Meng et al. further introduce additional Cre- and Dre-based genetic models. Notably, in control mice, skeletal muscle, an organ known to harbor a dense and well-characterized lymphatic network, shows no lymphatic labeling. This lack of signal indicates inefficient or incomplete recombination in tissues that are physiologically rich in lymphatics. In addition, in control mice lacking *Pik3ca*, the joint regions are systematically cropped from the images, precluding evaluation of lymphatic vessels in joints, which are among the most lymphatic-dense regions of the skeleton.

The dual recombinase model in Meng et al. is intrinsically pathological and characterized by abnormal cell proliferation. The tdTomato^+^ cells observed in this setting represent aberrant lymphatic hyperplasia rather than physiological lymphatic vessels, a conclusion further supported by distorted and disorganized architecture. Consistent with this interpretation, elevated LYVE1 expression and only LYVE1-high cells are *Prox1*^+^, indicating that *Prox1*-dependent recombination in this model selectively labels pathologically expanded abnormal lymphatic cells. Thus, *Prox1* labeling in this context reflects abnormal cellular expansion rather than normal lymphatic identity.

Importantly, while control mice lack detectable lymphatic labeling in skeletal muscle, *Pik3ca*-activated mice display robust lymphatic labeling in the same tissue. This stark contrast demonstrates that the genetic system lacks sensitivity for physiological lymphatics but selectively labels abnormal pathological lymphatic expansion. Consequently, observations derived from the *Pik3ca* model cannot be extrapolated to normal bone biology or used to argue against the presence of physiological bone lymphatics.

By contrast, multiple independent studies using physiological and injury-based models have established that bone lymphatics are present and functionally relevant *in vivo*. These vessels contribute to repair, regeneration, and immune remodeling. Enhancing lymphangiogenesis promotes bone formation. The *Pik3ca*-driven lymphatic hyperplasia associated with bone loss represents the opposite biological state. Therefore, the tdTomato^+^ LYVE1 high *Prox1*^+^ cells reported by Meng et al. reflect pathological lymphatic expansion and do not constitute evidence against the existence of bone lymphatics.

### Limitations in tissue clearing and imaging affect the interpretation of bone vascular architecture

The data presented rely on confocal microscopy of bone sections and lack the depth and anatomical continuity necessary to accurately assess physiological lymphatic and vascular architecture within the complex three-dimensional structure of bone. Only four low-resolution images are presented (Figures 1H, 1I, 2E, and 4B in Meng et al.), among which the positive control (Figure 1I, Meng et al.) lacks the epiphysis region.^28^ For this positive control, Figure 1I in Meng et al., they provide a 3D surface rendering of a *Cdh5-CreER; R26-tdTomato* mouse tibia in which the epiphyseal region is missing and the tdTomato signal is absent in regions of the bone. In this model, tdTomato should label ECs throughout the bone, given the high density of vessels. However, the conspicuous absence of tdTomato signal in substantial portions of the positive control and no epiphysis region of bone seen in this positive control indicates incomplete tissue clearing, limited antibody penetration, or inadequate imaging depth.

In another light-sheet image, Figure 1H in Meng et al., LYVE1 immunostaining is not detectable within the cortical bone or bone marrow, as confirmed by their own slice views and serial sections. Notably, Meng et al. themselves cite literature that documents LYVE1^+^ cells as residents of both bone marrow and cortical bone compartments. The complete absence of LYVE1^+^ staining in Figure 1H of Meng et al. therefore suggests insufficient tissue clearing, suboptimal antibody penetration, or limited imaging resolution. The two remaining light-sheet images in Figures 2E and 4B in Meng et al. are of POSTN, which does not contribute to the assessment of lymphatic vessels. Meng et al. provide two slice views, which further reinforce the technical limitations discussed above. Both views show that not only the epiphysis but also the metaphysis is truncated, directly contradicting the cleared-bone photographs. Importantly, these slice views do not demonstrate the presence of staining in other regions of the bone or across consecutive optical sections. The accompanying 3D rendering reveals discontinuous labeling, with large regions entirely devoid of vascular signal, indicative of incomplete tissue clearing, insufficient antibody penetration, or inadequate imaging depth.

Figure 4B in Meng et al. demonstrates likely issues with inadequate labeling or penetration rather than absence of lymphatics in bone, as in slice 255 and slice 350, where sparse tdTomato^+^ lymphatic vascular structures are detectable (Figure S4). This observation is consistent with the presence of only sporadic tdTomato^+^ vascular signals in joint regions in some images presented by Meng et al. Overall, the light-sheet data presented by Meng et al. suggest incomplete tissue clearing, limited penetration of labeling reagents, restricted imaging depth, and, in some cases, potential challenges in anatomical identification, which could influence the interpretation of their findings.

**Figure 4:**
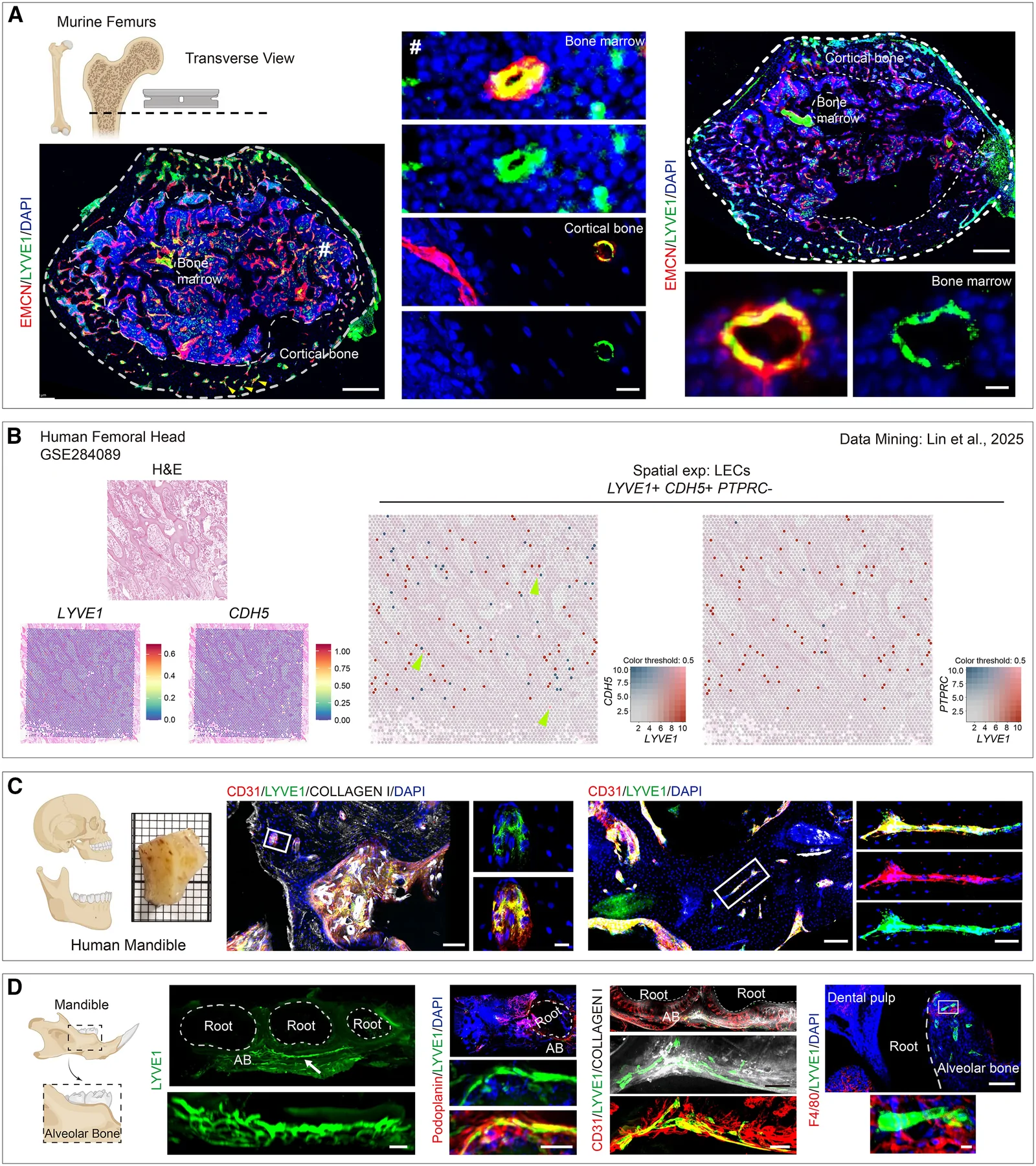
3D imaging of lymphatics within the bone and bone marrow microenvironment (A) Schematic representation of transverse imaging of murine femoral bones. Scale bars: 400 μm (overview) and 20 μm (insets). (B) Spatial transcriptomics analysis from a human femoral head. Green arrowheads indicate *LYVE1*^*+*^*CDH5*^*+*^*PTPRC*^*−*^ LECs. (C) 3D images showing immunolabeling for CD31, LYVE1, and collagen I. Scale bars: 300 μm (overview) and 20 μm (insets). (D) Light-sheet imaging of cleared alveolar bones from 10–12-week-old mice showing immunolabeling of LYVE1, podoplanin, CD31, collagen 1, and F4/80 and nuclear staining with DAPI. Scale bars: 400 μm (overview) and 20 μm (insets).

By contrast, our high-resolution light-sheet imaging demonstrates a robust LYVE1^+^ CD31^+^ signal co-localized within tubular vascular structures embedded in bone, which are morphologically distinct from LYVE1^+^ CD45^+^ round hematopoietic cells. We further extended our imaging analysis to include transverse views of murine femoral bones, which clearly show LYVE1^+^ and EMCN^+^ lymphatic vascular structures within both the bone marrow and cortical bone compartments (Figure 4A). To independently validate these findings, we analyzed publicly available spatial transcriptomics datasets from human femoral bone, which revealed the presence of LYVE1^+^ CDH5^+^ PTPRC^−^ LECs (Figure 4B). Similarly, 3D imaging of adult human mandibles revealed LYVE1^+^ CD31^+^ vascular structures in bone, as evidenced by collagen I immunostaining (Figure 4C). We visualize LYVE1^+^ and Podoplanin^+^ lymphatic vascular structures alongside CD31, as sinusoidal endothelium exhibits low CD31 expression. In addition, our light-sheet imaging of murine mandibles identifies LYVE1^+^ and Podoplanin^+^ vascular structures embedded within bone. These lymphatic vascular structures are negative for the macrophage marker F4/80 and positive for CD31 (Figure 4D). Taken together, our comprehensive, multimodal dataset, including light-sheet imaging of whole bones, spatial transcriptomics, and scRNA-seq, provides unequivocal evidence confirming the presence of LECs within bone. By contrast, Meng et al. do not detect LYVE1^+^ or Prox1^+^ structures, which reflects limitations in tissue processing, antibody penetration, and imaging methodology. High-resolution whole-bone imaging is essential for identifying sparse lymphatic vessels embedded within the dense bone matrix.

## Discussion

Our study provides multimodal evidence substantiating that lymphatic vessels are integral, spatially embedded in bone, and drive skeletal repair. By integrating high-resolution imaging, spatial transcriptomics, and scRNA-seq, it affirms the presence of LECs within bone and bone marrow. Our analyses demonstrate that the periosteum is an insufficient source of bone lymphatic endothelial cells and that bone LECs actively promote skeletal repair through regenerative lymphangiocrine signaling.

Lineage and cell-type classifications in scRNA-seq datasets are known to be context- and pipeline-dependent, and the field has not yet reached consensus on the definition of several bone and endothelial subsets. Meng et al. questioned the expression of *Clec14a*, a gene enriched in angiogenic endothelial subsets but is also expressed in LECs.^54^ Additionally, our original study indicated a lineage connection between lymphatic and specialized angiogenic type H bone endothelial populations. In line with this, our analysis of multiple publicly available datasets further confirms that *Clec14a* is expressed in both blood ECs and LECs (Figure S5).

No functional assays were performed. The animal model lacks detection of LECs in well-established lymphatic-rich areas such as joints and knees is likely attributable to multiple factors, including the recombination protocol, reporters, and detection approaches required to visualize spatially complex tissues like bone. However, optimization of protocols and sequential troubleshooting approaches of critical steps would enable visualizing the detailed structures within the marrow cavity. The authors rely heavily on confocal imaging of bone sections and provide four countable images of whole/intact-bone light-sheet imaging, which demonstrate incomplete tissue clearing and inadequate antibody penetration. Albeit with the caveat that spatial transcriptomics constrains single-cell resolution, our spatial transcriptomic analyses reveal consistent and reproducible localization of *Prox1*^+^ LECs within both cortical bone and the marrow cavity. Our scRNA-seq datasets, spanning murine and human bone from embryonic development through adulthood, robustly validate the presence of PROX1^high^ LECs across multiple anatomical sites, with frequencies comparable to or exceeding those observed in soft-tissue endothelial atlases where LEC populations are well established.

Together, the convergence of evidence from imaging and transcriptomic profiling underscores a central biological principle: lymphatic vessels are an integral component of bone architecture. Light-sheet microscopy of intact bones is essential in resolving sparse and spatially restricted sparse lymphatic structures in mineralized tissues that are routinely overlooked by conventional methods. Our imaging with morphological resolution clearly distinguishes LYVE1^+^ lymphatic vessels embedded within bone from LYVE1^+^ CD45^+^ hematopoietic cells. Lymphatic dysfunction drives osteonecrosis of the jaw, impairs fracture repair, and accelerates periprosthetic bone loss. Enhancing lymphangiogenesis restores bone formation, and lymphangiogenic biomaterials, VEGF-C delivery, and JAK inhibition represent tractable therapeutic strategies for osteoporosis, osteonecrosis, and impaired skeletal repair.

Our analyses consistently and reproducibly identify Prox1^+^ LECs across multiple independent murine and human datasets spanning embryonic development through adulthood, across diverse anatomical sites, sequencing platforms, and tissue processing workflows. Resolving this question carries direct therapeutic relevance: lymphatic vessels regulate fracture repair, limit inflammatory bone loss, and modulate periprosthetic osteolysis through VEGF-C/VEGFR3 signaling, and their dysfunction contributes to bisphosphonate-related osteonecrosis of the jaw. In conclusion, our study provides convergent, reproducible, and multimodal evidence that LECs are an integral and functionally significant component of the bone microenvironment.

### Limitations of the study

Single-cell transcriptomic analyses are inherently limited by dissociation biases that can underrepresent sparse or embedded populations such as LECs. This challenge is not unique to bone: even in soft-tissue endothelial atlases from lymphatic-rich organs (e.g., skeletal muscle), *Prox1*^+^ cells often remain sparse or absent. These shared technical constraints suggest that difficulties in LEC detection reflect general limitations of the methodology. Likewise, spatial transcriptomics preserves anatomical context but remains constrained by resolution and gene detection sensitivity, potentially limiting the detection of low-abundance transcripts and closely juxtaposed cell populations. Although our conclusions are supported by multiple murine and human datasets, variability in anatomical sampling, sequencing depth, and cell recovery introduces heterogeneity in LEC detection. This is particularly evident in human datasets, where low endothelial representation restricts comparisons across samples and conditions.

## Resource availability

### Lead contact

Further information and requests for resources and reagents should be directed to and will be fulfilled by the lead contact, Anjali P. Kusumbe (anjali.pkusumbe@ntu.edu.sg).

### Materials availability

This study did not generate any new, unique reagents.

### Data and code availability

All codes used for the analysis of single-cell sequencing and spatial transcriptomics data are provided in Methods S1.

## Acknowledgments

This work is supported by the Ministry of Education (MOE) Singapore, MOE—Academic Research Funds (#024983-00001 and #025277-00026); the European Research Council (StG: metaNiche, 805201); and the European Union’s Horizon 2020 (no. 857524) to A.P.K. S.K.R. is supported by the Ministry of Education (MOE RS13/24) Singapore and the European Union’s Horizon 2020. J.C. is supported by the National Nature Science Foundations of China (grant nos. 82422021 and 82270961) and the Sichuan Science and Technology Program (grant no. 2023JDRC0018). S.J. is supported by the Nanyang Technological University Research Scholarship (reg. no. 200604393R). Y.Y. is supported by the National Nature Science Foundations of China (grant no. 824B2026).

## Author contributions

Y.Y. designed and performed bioinformatics analyses and assembled figures. Y.Z. assembled figures. J.C., Y.S., and J.D.J. designed and performed imaging experiments and analyzed data. S.J. designed and performed single-cell sequencing analyses and assembled figures. Y.Y., S.J., and J.C. prepared figures and revised the manuscript. A.S. contributed to single-cell sequencing analyses. S.K.R. contributed to data analysis. A.P.K. designed and directed the study, analyzed data, and wrote the manuscript.

## Declaration of interests

The authors declare no competing interests.

## STAR★Methods

### Key resources table

**Table:** 

REAGENT or RESOURCE	SOURCE	IDENTIFIER
**Antibodies**		

Endomucin	Santa Cruz Biotechnology	CAT#sc-65495; RRID: AB_2100037
CD31	R&D Systems	CAT#AF3628; RRID: AB_2161028
Collagen1	Sigma-Aldrich	CAT#AB765P RRID: AB_92259
LYVE1	Abcam	CAT#ab14917; RRID: AB_301509
Podoplanin	R&D Systems	CAT#AF3244; RRID: AB_2268062
F4/80	Bio-Rad	CAT#MCA497GA; RRID: AB_323806
DACH1	Proteintech	CAT#10914-1-AP; RRID: AB_2230330
Osteopontin	R&D Systems	CAT#AF808; RRID: AB_2194992
Runx2	R&D Systems	CAT#MAB2006; RRID: AB_2184526
Donkey anti-Rabbit IgG (H + L) Alexa Fluor 488	ThermoFisher Scientific	CAT#A-21206; RRID: AB_2535792
Donkey anti-Rabbit IgG (H + L) Alexa Fluor 647	ThermoFisher Scientific	CAT#A-31573; RRID: AB_2536183
Donkey anti-Rabbit IgG (H + L) Alexa Fluor 594	ThermoFisher Scientific	Cat# A-21207, RRID: AB_141637
Donkey anti-Rat IgG (H + L) Alexa Fluor 488	ThermoFisher Scientific	CAT#A-21208; RRID: AB_2535794
Donkey anti-Rat IgG H&L (Alexa Fluor 647)	Invitrogen	CAT#A48272; RRID: AB_2893138
Donkey anti-Goat IgG (H + L) Alexa Fluor 488	ThermoFisher Scientific	CAT#A-11055; RRID: AB_2534102
Donkey anti-Goat IgG (H + L) Alexa Fluor 647	ThermoFisher Scientific	CAT#A-21447; RRID: AB_2535864
Donkey anti-Goat IgG (H + L) Alexa Fluor 546	ThermoFisher Scientific	CAT#A-11056; RRID: AB_2534103

**Biological samples**		

Human mandibular bone specimens obtained from osteotomy patients	West China Hospital of Stomatology, Sichuan University (Chengdu, China)	N/A

**Chemicals, peptides, and recombinant proteins**		

Ethanol	Sigma-Aldrich	CAT#1070172511
Paraformaldehyde	Sigma-Aldrich	CAT#158127
PBS (10X)	VWR	CAT#437117K
PBS Tablets	Biobasic	CAT#PD0435
EDTA	Sigma	CAT#E6758
Methanol	Sigma-Aldrich	CAT#179337
Hydrogen Peroxide	Sigma-Aldrich	CAT#HX0636
Urea	Sigma-Aldrich	CAT#U5128
Glycerol	Sigma-Aldrich	CAT#G7893
Triton X-100	Sigma-Aldrich	CAT#T8787
Donkey serum	Solarbio,	CAT#SL050
Sucrose	Sigma-Aldrich	CAT#V900116
Gelatin	Sigma-Aldrich	CAT#G2625
Polyvinylpyrrolidone	Sigma-Aldrich	CAT# P5288
O.C.T	VWR	CAT#361603E
DAPI	Solarbio	CAT# C0060
Fluoromount-G	Invitrogen	CAT#00-4958-02
Collagenase	Merck	CAT#10103578001
Fetal Bovine Serum	Sigma-Aldrich	CAT#F7524
DMSO	Sigma-Aldrich	CAT#D5879
Isopropanol	Sigma-Aldrich	CAT#190764
Ethyl cinnamate (ECi)	Sigma-Aldrich,	CAT#112372
PEGM	Sigma-Aldrich	CAT#447943

**Deposited data**		

Analysis Codes	GitHub repository	https://github.com/sunny-yangyang/Multimodal.git

**Experimental models: Organisms/strains**		

Mouse: C57BL/6J	Charles river	Strain#632

**Software and algorithms**		

GraphPad Prism (version 10.3.1)	GraphPad Software, La Jolla, CA, USA	https://www.graphpad.com
R Studio (version 4.5.2)	Posit PBC	https://cran.r-project.org
Imaris (version 10.2.0)	Bitplane	https://imaris.oxinst.com/packages
Imaris File converter (version 10.2.0)	Bitplane	https://imaris.oxinst.com/packages
Zen Black (version 3.1)	Zeiss (Jena, Germany)	https://www.micro-shop.zeiss.com/en/us/softwarefinder/software-categories/zen-black/zen-black-system/
Adobe Photoshop CC	Adobe	https://www.adobe.com/uk/products/photoshop
Adobe Illustrator CC	Adobe	https://www.adobe.com/uk/products/illustrator

**Others**		

Low-profile blades	Leica	CAT#14035838382
Zeiss Laser scanning confocal microscope LSM880	Carl Zeisis AG	N/A
Zeiss Lightsheet 7 microscope	Carl Zeisis AG	N/A
Water bath	Stuart	CAT#SBS40
Leica CM1950 cryostat	Leica Co., Germany	N/A
Microscopic Slides	ThermoFisher Scientific	CAT#1237-3118
Disposable Base molds	Ted Pella. INC	CAT#27147-2
Disposable Base molds	Ted Pella. INC	CAT#27147-4
Cover Slips	VWR	CAT#631-0135

### Method details

#### scRNA-seq analysis

Quality control (QC) metrics, including the number of Single-cell RNA sequencing (scRNA-seq) data were obtained from the Gene Expression Omnibus (GEO) under accession number. Raw count matrices were downloaded in 10X Genomics output format and imported into R (v4.4.1) using the Read10X function from the Seurat package (v5.2.1,^5^ DOI: 10.1038/s41587-023-01767-y). Individual Seurat objects were created with CreateSeuratObject and merged using the merge function to construct a unified dataset. Sample metadata was retained to distinguish experimental groups.

Genes (nFeature_RNA), total UMI counts (nCount_RNA), and the proportion of mitochondrial genes (identified using PercentageFeatureSet with "mt-/MT-" prefix), were calculated. Cells were retained if nCount_RNA > 1000, nFeature_RNA > 500, and mitochondrial content was less than 10%. QC distributions were visualized using VlnPlot from Seurat.

Data normalization was performed using NormalizeData (method = "LogNormalize"), followed by scaling with ScaleData. Highly variable genes (n = 2000) were selected via FindVariableFeatures using the "vst" method, and principal component analysis (PCA) was applied using RunPCA. The number of informative dimensions was selected based on an elbow plot (ElbowPlot), and the first 25 principal components were used to construct a shared nearest neighbor graph (FindNeighbors).

Batch correction across samples was conducted using Harmony (v1.2.3; Korsunsky et al., DOI: 10.1038/s41592-019-0619-0), applied via RunHarmony with sample identity as the covariate. Non-linear dimensionality reduction was performed using UMAP, applied on Harmony-corrected dimensions.

Clusters were identified using FindClusters across multiple resolutions and visualized using UMAP. Cluster resolution and stability were assessed using clustree (v0.5.1; Zappia & Oshlack, DOI: 10.1093/gigascience/giy083). Endothelial cells were manually annotated based on canonical marker gene expression, visualized with FeaturePlot and VlnPlot functions.

The endothelial cell population was extracted based on cluster annotations and re-subclustered by reprocessing with PCA, clustering, and visualization. Accurate resolution and the top 20 PCs were used. Prox1 expression values were retrieved using FetchData and classified into Prox1+ and Prox1- subclusters using a threshold set at two standard deviations above the mean expression.

Gene expression visualization was performed using DotPlot, FeaturePlot, and VlnPlot to highlight marker genes and assess transcriptional heterogeneity. Relative cluster proportions between conditions were calculated using reshape2 (v1.4.4). Figures were rendered using ggplot2 (v3.5.1; Wickham, DOI: 10.1007/978-3-319-24277-4_9) and patchwork (v1.3.0).

#### Spatial transcriptomics analysis

Spatial transcriptomics data were processed using Seurat (v5.2.1,^5^ DOI: 10.1038/s41587-023-01767-y) in R v4.4.1. Raw data generated by 10X Genomics Visium technology were imported using the Load10X_Spatial function, with the input consisting of a filtered.h5 expression matrix and accompanying spatial directory.

The Seurat object was initialized with project-level metadata (project.name, orig.ident). Quality control was conducted by visualizing spatial transcript counts (nCount_Spatial) using violin plots (VlnPlot) and tissue overlays (SpatialFeaturePlot).

Gene expression normalization was performed using SCTransform, applied to the "Spatial" assay. Dimensionality reduction was performed using PCA via RunPCA, followed by visualization with DimPlot and ElbowPlot to determine the number of informative PCs.

Clustering was carried out using the shared nearest neighbor (SNN) approach on the top 15 principal components with FindNeighbors and FindClusters. UMAP embedding was computed using RunUMAP, and clusters were visualized both in UMAP space (DimPlot) and tissue context (SpatialDimPlot).

To assess tissue-level spatial structure, raw spatial coordinates were parsed from tissue positions list, with in-tissue barcodes retained. These were aligned with Seurat metadata to generate 2D scatterplots via ggplot2 (v3.5.1; Wickham, DOI: 10.1007/978-3-319-24277-4_9). Spatial coordinates were also embedded into the Seurat object as a new dimensionality reduction (CreateDimReducObject), stored as "space".

Gene expression features such as *Prox1* and *Ptprc* were visualized across spatial domains using FeaturePlot and SpatialFeaturePlot. Blended expression plots were generated with blend = TRUE, using custom color scales and thresholds. Analysis codes are provided in Methods S1.

#### Mice

The study employed C57BL/6 mice from Charles River as wild-type subjects for all analyses, unless otherwise specified. Both male and female mice were used. The study adhered to the Principles of Laboratory Animal Care outlined by the National Society for Medical Research and the Guide for the Care and Use of Laboratory Animals (National Academies Press, 2011). All Experiments throughout the study involving animals were performed according to institutional guidelines and laws, following protocols approved by local animal ethics committees of Nanyang Technological University, Singapore, West China Hospital of Stomatology, Sichuan University and University of Oxford, UK and conducted in compliance with institutional guidelines.

#### Human samples

Human mandibular bone specimens were obtained from osteotomy patients in March 2025 at West China Hospital of Stomatology, Sichuan University (Chengdu, China). All samples were collected intraoperatively by experienced oral and maxillofacial surgeons. The study protocol was approved by the Institutional Review Board of West China Hospital of Stomatology. Written informed consent was obtained from all participants prior to sample collection.

#### Bone collection, fixation, and decalcification

Freshly dissected bone tissues were fixed in ice-cold 4% PFA for 2.5 hours after removal of all soft tissues. Decalcification was performed using 0.5 M EDTA at room temperature for 2 days with twice-daily buffer exchanges. After PBS washes, bones were dehydrated through a graded methanol (Sigma-Aldrich, 179337) series by immersion in ice-cold 50% and 80% methanol for 30 minutes each, followed by 100% methanol for 1 hour with two changes of fresh methanol every 20 minutes. Tissues were then bleached in 5% (v/v) hydrogen peroxide (H₂O₂; Sigma-Aldrich, HX0636) for 1 hour at room temperature. After bleaching, samples were rehydrated through a descending methanol gradient and washed in PBS three times for 20 minutes each.

#### Sample preparation for immunostaining

Decalcified bones were incubated in 20% sucrose (Sigma-Aldrich, V900116) + 2% polyvinylpyrrolidone (PVP, Sigma-Aldrich, P5288) at 4°C for 12 hours, embedded in 8% gelatin (Sigma-Aldrich, G1890) with 20% sucrose and 2% PVP, and cryosectioned into 70-μm slices using a Leica CM1950 cryostat (Leica Co., Germany). Sections were air-dried and stored at -20°C.

#### Immunostaining on thick bone slices

Sections were rehydrated, permeabilized with 0.3% Triton X-100, and blocked with 5% donkey serum. Primary antibodies were applied at 1:150 dilution for 4 hours at room temperature, followed by Alexa Fluor–conjugated secondary antibodies (1:300) for 1–1.5 hours. Nuclei were stained with DAPI (Solarbio, C0060) and mounted using Fluoromount-G (Invitrogen, 00-4958-02). Slides were stored at 4°C until imaging.

#### Whole bone immunolabeling

Antigen retrieval and permeabilization were performed by incubating bones in a solution containing 25% (w/v) urea (Sigma-Aldrich, U5128), 15% (w/v) glycerol (Sigma-Aldrich, G7893), 15% (v/v) Triton X-100 (Sigma-Aldrich, T8787), and 45% (v/v) double-distilled water at 4°C for 5 hours. Tissues were then treated with 0.2% collagenase (Merk, 10103578001) at 37°C for 30 minutes. After PBS washes containing 2% FBS, samples were blocked in PBS with 10% donkey serum (Solarbio, SL050), 10% DMSO, and 0.5% Triton X-100 at 37°C for 20 minutes. Primary antibodies were applied in antibody dilution buffer (2% donkey serum, 10% DMSO, 0.5% Triton X-100 in PBS) and incubated overnight. After primary antibody incubation, samples were extensively washed in PBS containing 2% donkey serum and 0.5% Triton X-100 at 37°C with shaking at 70 rpm for 3 hours. During the first hour, the wash buffer was replaced every 15 minutes, followed by changes every 30 minutes thereafter. Tissues were then incubated with fluorophore-conjugated secondary antibodies diluted in antibody dilution buffer for 6–8 hours at 37°C under gentle agitation. The same wash protocol as for the primary antibodies was applied to remove unbound antibodies. Antibody information is provided in the key resources table.

#### Dehydration and clearing of whole bones

Following immunolabeling, samples were dehydrated in a graded isopropanol (Sigma-Aldrich, 190764) series (30%, 50%, 80%) for 30 minutes each, followed by incubation in 100% isopropanol for 1 hour (replaced every 20 minutes). Samples were cleared in ECi (Sigma-Aldrich, 112372) and then equilibrated in 80% ECi + 20% PEGM (Sigma, 447943) clearing solution. Finally, specimens were stored in ECi at 4°C.

#### Light sheet imaging set-up and image acquisition

Cleared samples were imaged using a Zeiss Lightsheet 7 microscope with Zen Black (v3.1) software and a 5× EC Plan-Neofluar objective. Excitation wavelengths included 405, 488, 561, and 639 nm. Samples were affixed to the sample holder using cyanoacrylate and immersed in ECi (*n* = 1.57) during imaging. Images were converted using Imaris File Converter (v10.2.0) and analyzed using Imaris (v10.2.0).

## Supplemental information

Document S1. Figures S1–S5

Methods S1. Single-cell RNA sequencing and spatial transcriptomic analyses, related to STAR Methods

Document S2. Article plus supplemental information
