# Supplementary material for: Multimodal evidence for bone lymphatics in skeletal health and repair
Source: Cell. 2026 Aug 20;189(17):5466–5477.e4. doi: 10.1016/j.cell.2026.06.026 (PMC13502997; doi:10.1016/j.cell.2026.06.026)
Supplement: Supplementary file 1 [file mmc1.pdf]

Cell, Volume *189*

Supplemental information

Multimodal evidence for bone lymphatics

in skeletal health and repair

Yang Yang, Yuheng Zhang, Yixin Shi, Sanyam Jain, Jessy D. Joseph, Amit Singh, Junyu Chen, Saravana K. Ramasamy, and Anjali P. Kusumbe

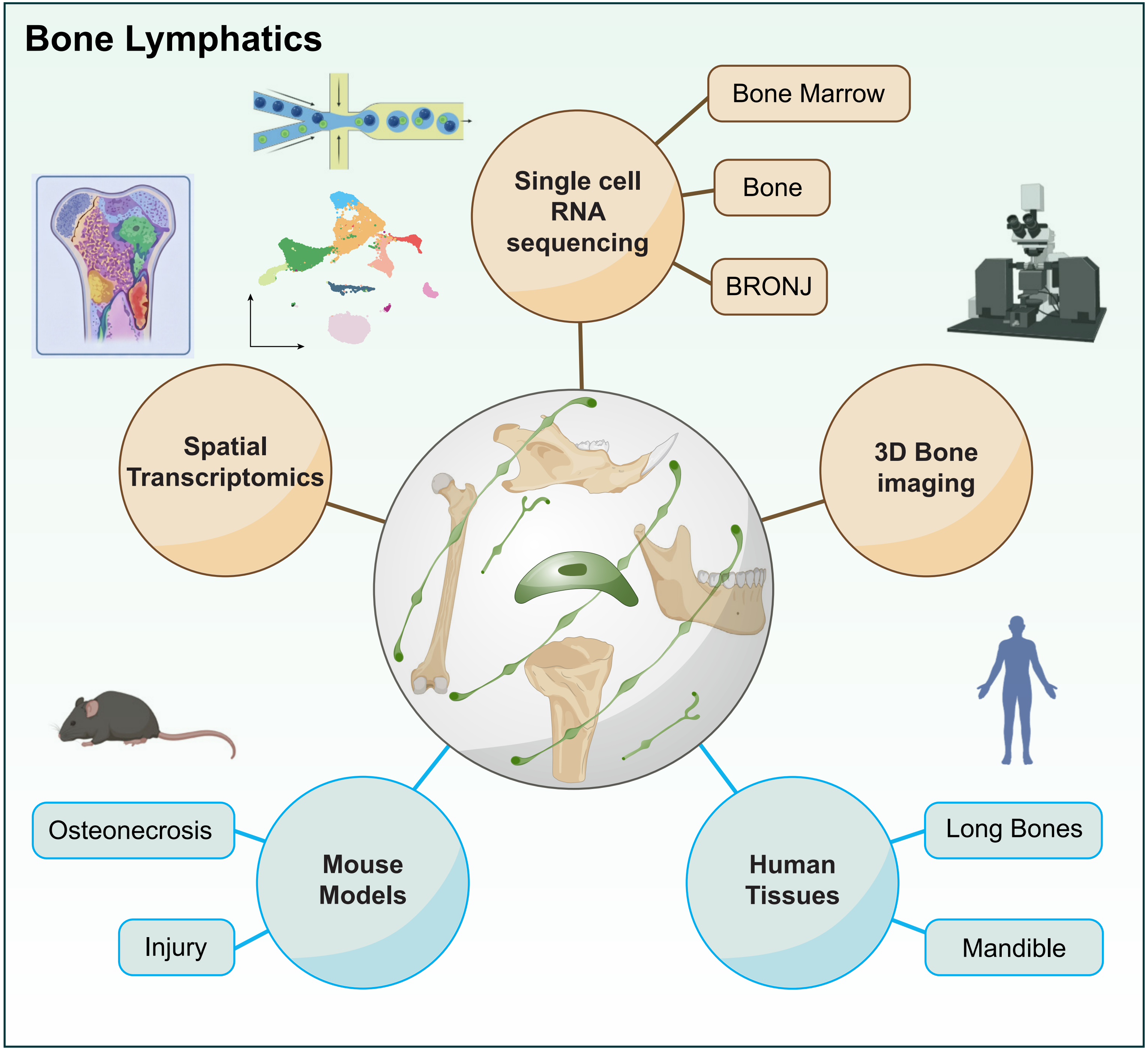

Figure S1. Schematic representing the experimental analyses (Related to Figure 1 and STAR METHODS)

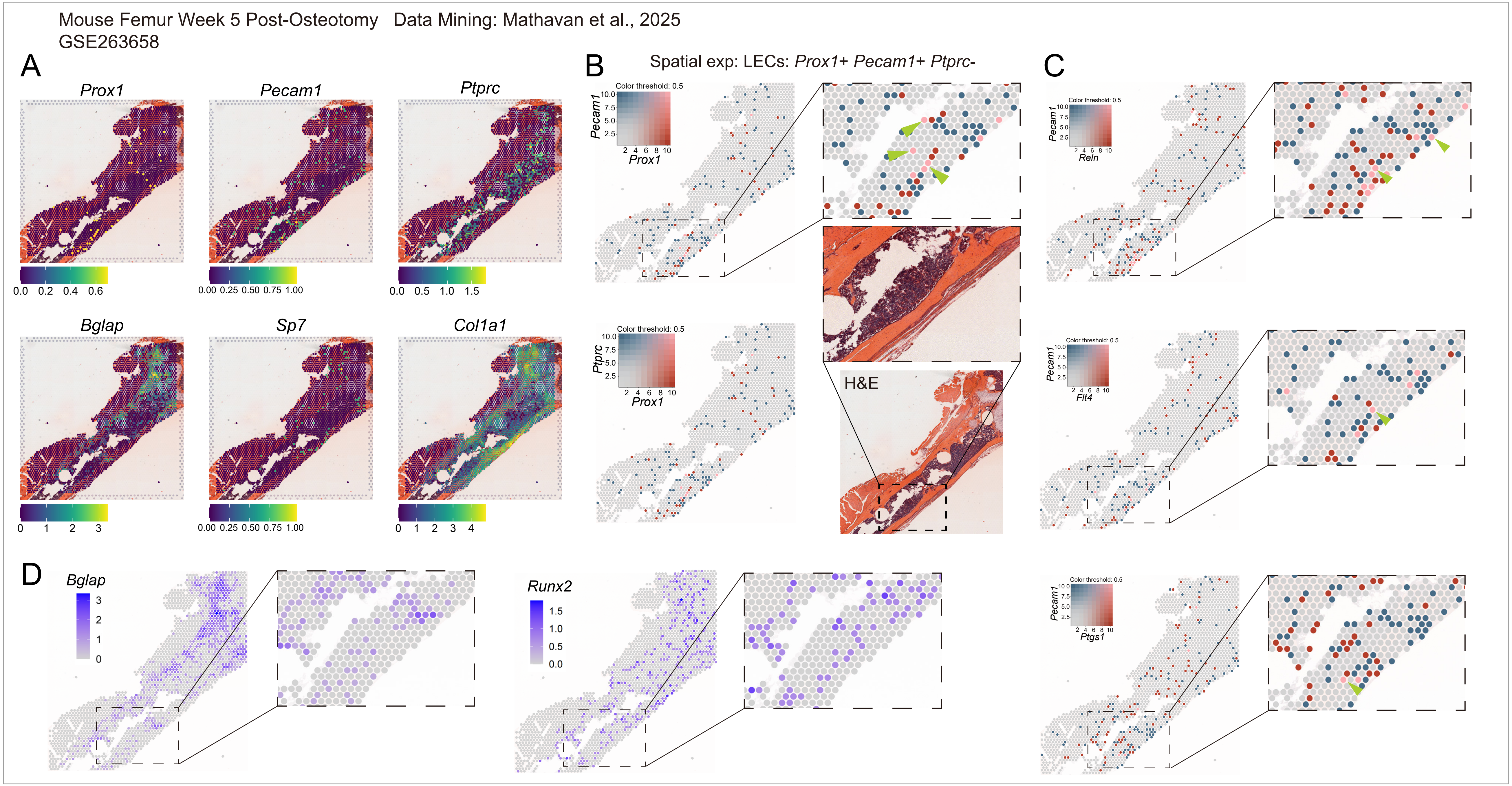

Figure S2. Spatial analysis of lymphatic endothelial cells in bones (Related to Figure 1)

Spatial transcriptomics data from Mathavan et al., 2025, using a femur from BCR reporter mice after bridged osteotomy with 0 N sham-loading. (A) Spatial feature plots show the expression of *Prox1*, *Pecam1*, *Ptprc*, *Bglap*, *Sp7*, and *Col1a1*. (B) Co-expression of *Prox1* with *Pecam1* and *Ptprc* is visualized. Green arrowheads indicate *Prox1*⁺ *Pecam1*⁺ *Ptprc*⁻ LECs in the cortical bone and marrow. H&E staining provides a histological context. (C) Co-expression of *Reln*⁺ *Pecam1*⁺, *Flt4*⁺ *Pecam1*⁺, and *Ptgs1*⁺ *Pecam1*⁺ is visualized. Green arrowheads indicate double-positive cells in the cortical bone and marrow. H&E staining provides a histological context. (D) Feature plots show the expression of *Bglap* and *Runx2*.

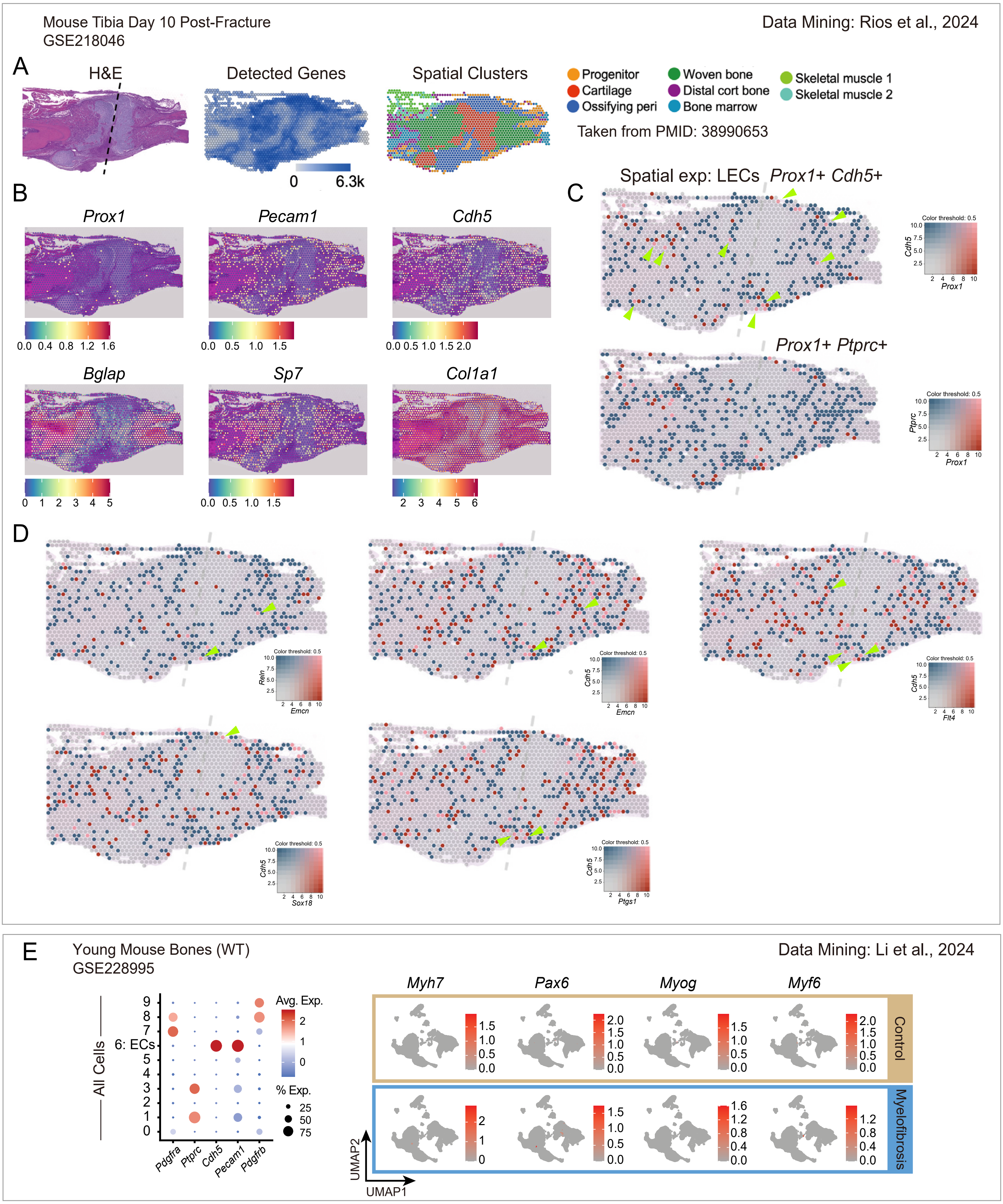

Figure S3. Spatial transcriptomics analysis of bone lymphatic endothelial cells (Related to Figure 1)
Spatial transcriptomics data from Rios et al. (2024), analyzed from the fracture callus of *Nf1+/fl* mice at day 10 post-injury. (A) H&E-stained spatial histology and maps of detected gene counts or computationally defined cell clusters are shown. Dashed line indicates the fracture plane (taken from PMID: 38990653). (B) Spatial feature plots display *Prox1*, *Pecam1*, *Cdh5*, *Bglap*, *Sp7*, and *Col1a1* expression. (C) Co-expression of Prox1 with *Pecam1* and *Ptprc* is visualized. Green arrowheads indicate *Prox1*$^+$ *Cdh5*$^+$ *Ptprc*$^-$ LECs in the bone and bone marrow. (D) Co-expression of *Reln*$^+$*Cdh5*$^+$, *Emcn*$^+$*Cdh5*$^+$, *Flt4*$^+$*Cdh5*$^+$, *Sox18*$^+$*Cdh5*$^+$ and *Ptgs1*$^+$*Cdh5*$^+$ is visualized. Green arrowheads indicate double-positive cells in the cortical bone and marrow. H&E staining provides a histological context. (E) scRNA-seq data from Li et al. (2024), profiling bone marrow stromal cells from young wild-type (WT) mice. Dot plot illustrates the distribution of stromal cell populations, including endothelial cells (ECs). Feature plots show that myogenic genes including *Myh7*, *Pax6*, *Myog*, and *Myf6* are not detectably expressed in either group.

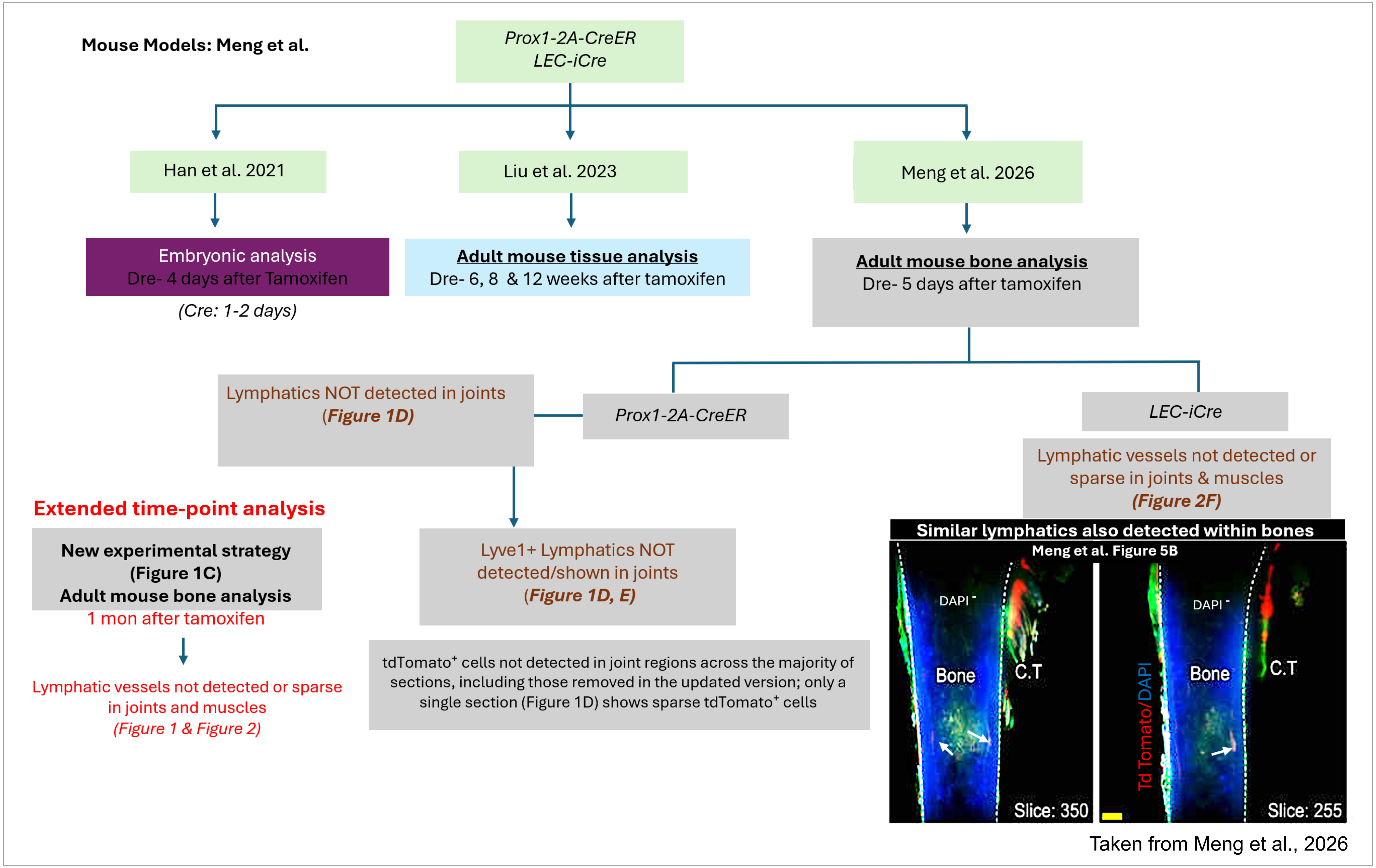

Figure S4. Mouse models used by Meng et al. do not detect lymphatics in joints (Related to Figure 4)
In Han et al. (2021) embryonic tissues were analyzed 4 days post-induction, compared to 1-2 days induction required for Cre in embryo. In their previous study where they have employed this mouse model Liu et al. 2023 examined adult tissues 6–12 weeks after tamoxifen induction. In contrast, Meng et al.'s analysis of bone tissues in adult mice 5 days post-induction lacks justification and deviates from the established protocols. These models do not detect lymphatics in joints as evident in Meng et al.'s Figure 1D and in LEC-iCre Figure 2F of Meng et. al no detection of lymphatics in joints and muscles.

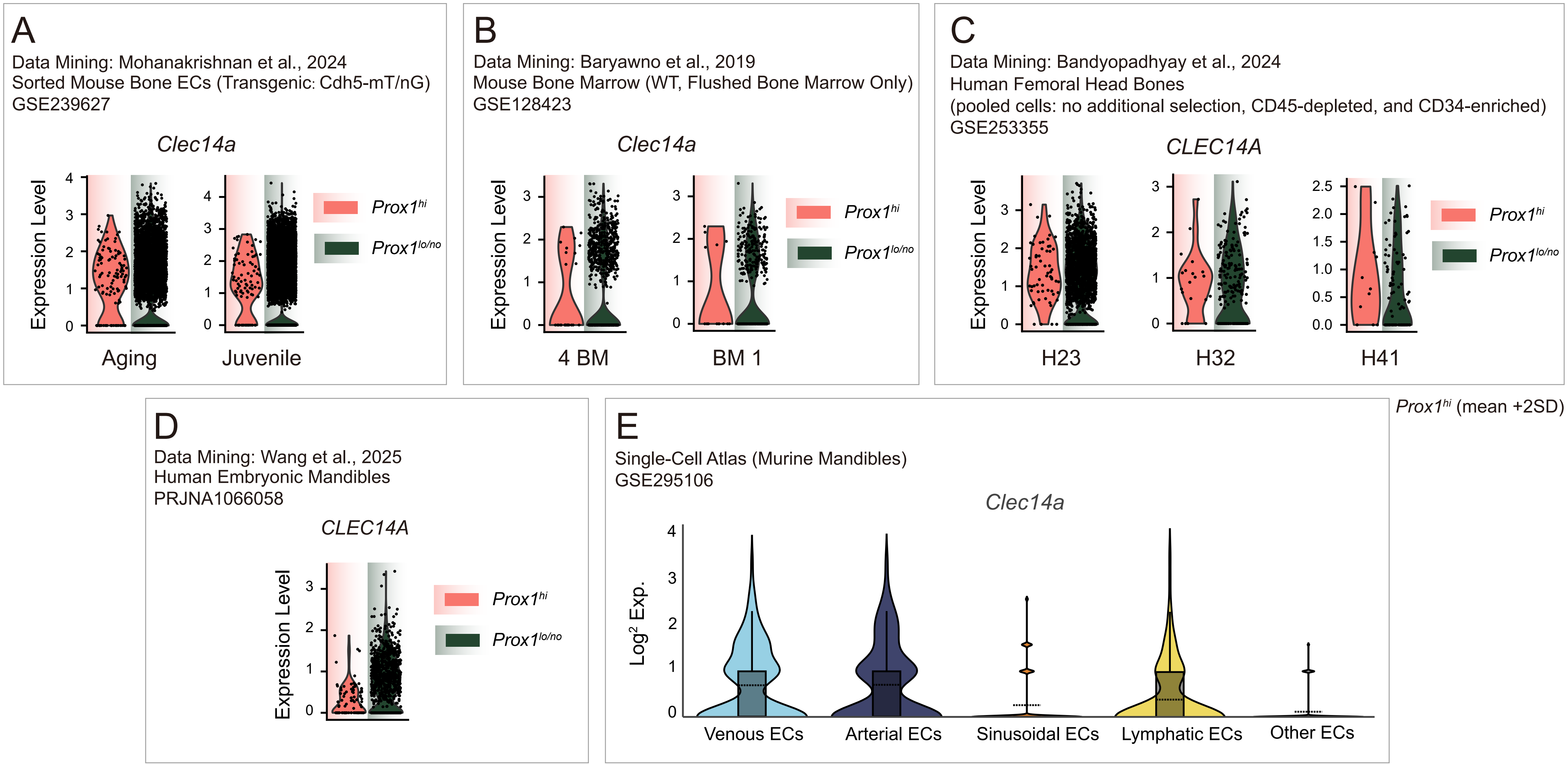

Figure S5. *Clec14a* expression in bone lymphatic endothelial cells (Related to Figure 2 and 3)
(A-E) Violin plots showing *Clec14a* expression in both ECs and LECs across multiple datasets.
