# Supplementary material for: Multimodal evidence for bone lymphatics in skeletal health and repair
Source: Cell. 2026 Aug 20;189(17):5466–5477.e4. doi: 10.1016/j.cell.2026.06.026 (PMC13502997; doi:10.1016/j.cell.2026.06.026)
Supplement: Supplementary file 2 [file mmc2.pdf]

Methods S1: Code Availability: Single-Cell RNA sequencing and Spatial Transcriptomic Analyses (Related to STAR Methods)

Single Cell-RNA sequencing analysis (Lymphatic Endothelial Cells)

```
# Load required packages
library(Matrix)
library(devtools)
library(harmony)
library(multtest)
library(Seurat)
library(dplyr)
library(patchwork)
library(R.utils)
library(ggplot2)
library(SeuratData)
library(magrittr)
library(clustree)
library(reshape2)

# Set working directory and read data setwd("F:/Data analysis")
seurat_data1 <- Read10X(data.dir = "GSE190965/")
seurat_data2 <- Read10X(data.dir = "GSE190966/") # Placeholder
seurat_data3 <- Read10X(data.dir = "GSE190967/") # Placeholder

# Create Seurat objects
seurat_obj1 <- CreateSeuratObject(counts = seurat_data1, project = "sample1")
seurat_obj2 <- CreateSeuratObject(counts = seurat_data2, project = "sample2")
seurat_obj3 <- CreateSeuratObject(counts = seurat_data3, project = "sample3")

# Merge all Seurat objects
seurat_obj <- merge(x = seurat_obj1, y = list(seurat_obj2, seurat_obj3))

# Quality Control
seurat_obj[["percent.mt"]] <- PercentageFeatureSet(seurat_obj, pattern = "^mt-")
VlnPlot(seurat_obj, features = c("nFeature_RNA", "nCount_RNA", "percent.mt"), ncol = 3,
        pt.size = 0.1)

# Filter cells
seurat_obj <- subset(seurat_obj,
subset = nCount_RNA > 1000 & nFeature_RNA > 500 & percent.mt < 10)
VlnPlot(seurat_obj, features = c("nFeature_RNA", "nCount_RNA", "percent.mt"), ncol = 3,
        pt.size = 0.1)
```

```r
# Normalization & Dimension Reduction
seurat_obj <- NormalizeData(seurat_obj)
seurat_obj <- FindVariableFeatures(seurat_obj, selection.method = "vst", nfeatures = 2000)
seurat_obj <- ScaleData(seurat_obj) seurat_obj <- RunPCA(seurat_obj, features =
VariableFeatures(seurat_obj), verbose = FALSE) DimPlot(seurat_obj, reduction = "pca")

# Harmony batch correction
seurat_obj <- RunHarmony(seurat_obj, "orig.ident")

# Elbow plot for PC selection
ElbowPlot(seurat_obj)

# Clustering & non-linear reduction
seurat_obj <- FindNeighbors(seurat_obj, dims = 1:25) seurat_obj <- RunUMAP(seurat_obj,
                                dims = 1:25, reduction = "harmony")
seurat_obj <- RunTSNE(seurat_obj, dims = 1:25, reduction = "harmony")
seurat_obj <- FindClusters(seurat_obj, resolution = 0.5)

# Check multi-resolution if needed
# clustree(seurat_obj@meta.data, prefix = "RNA_snn_res.")


# Cluster Annotation
seurat_obj$firstcelltype <- NA
seurat_obj$firstcelltype[seurat_obj$seurat_clusters == 3] <- "Endothelial"
seurat_obj$firstcelltype[seurat_obj$seurat_clusters == 1] <- "PDGFRA+"
seurat_obj$firstcelltype[seurat_obj$seurat_clusters == 7] <- "PDGFRB+"
seurat_obj$firstcelltype[seurat_obj$seurat_clusters == 6] <- "PDGFRA+PDGFRB+"
seurat_obj$firstcelltype[seurat_obj$seurat_clusters == 0] <- "C1"
seurat_obj$firstcelltype[seurat_obj$seurat_clusters == 2] <- "C2"
seurat_obj$firstcelltype[seurat_obj$seurat_clusters == 4] <- "C3"
seurat_obj$firstcelltype[seurat_obj$seurat_clusters == 5] <- "C4"
seurat_obj$firstcelltype[seurat_obj$seurat_clusters == 8] <- "C5"
seurat_obj$firstcelltype[seurat_obj$seurat_clusters == 9] <- "C6"

seurat_obj$firstcelltype <- factor(seurat_obj$firstcelltype,
levels = c("C1", "C2", "C3", "C4", "C5", "C6", "Endothelial", "PDGFRA+",
                                "PDGFRA+PDGFRB+", "PDGFRB+"))

# Plot marker expression
VlnPlot(seurat_obj, features = c("Prox1","Pecam1","Cdh5","Ptptc"), pt.size = 0.1, group.by =
                                "firstcelltype")
```

```
# Subset and Re-analysis
Endothelial <- subset(seurat_obj, subset = firstcelltype == "Endothelial")
Endothelial <- NormalizeData(Endothelial)
Endothelial <- FindVariableFeatures(Endothelial)
Endothelial <- ScaleData(Endothelial)
Endothelial <- RunPCA(Endothelial, features = VariableFeatures(Endothelial), verbose =
                                   FALSE)
ElbowPlot(Endothelial)
Endothelial <- FindNeighbors(Endothelial, dims = 1:20)
Endothelial <- FindClusters(Endothelial, resolution = 0.4) # Check multi-resolution if needed
# clustree(seurat_obj@meta.data, prefix = "RNA_snn_res.")
Endothelial <- RunTSNE(Endothelial, dims = 1:20)
Endothelial <- RunUMAP(Endothelial, dims = 1:20)

# Visualize clustering
DimPlot(Endothelial, reduction = "tsne")
DimPlot(Endothelial, reduction = "umap")

# Prox1hi Classification
gene_expression <- FetchData(Endothelial, vars = "Prox1")
threshold <- mean(gene_expression$Prox1, na.rm = TRUE) + 2 *
sd(gene_expression$Prox1, na.rm = TRUE)
Endothelial$Prox1_Classification <- ifelse(gene_expression$Prox1 > threshold, "Prox1hi",
                                   "Prox1low")

# Plots
DotPlot(Endothelial, features = c("Pdgfra", "Ptprc", "Cdh5", "Pecam1", "Pdgfrb")) +
theme(axis.text.x = element_text(angle = 45, hjust = 1)) + scale_color_gradientn(colors =
                                   c("#4874CB", "white", "#C2000A"))
FeaturePlot(Endothelial, features = "Pax6", pt.size = 0.5, reduction = "umap", cols =
                                   c("lightgrey", "red"))
VlnPlot(Endothelial, features = "Prox1", pt.size = 0.1, group.by = " Prox1_Classification")


# Count table
df <- as.data.frame(table(RE_Endothelial@meta.data$group,
                                   RE_Endothelial@meta.data$secondcelltype))
colnames(df) <- c("group", "secondcelltype", "Freq")
df_wide <- dcast(df, secondcelltype ~ group, value.var = "Freq", fill = 0)
write.table(df_wide, file = "group_number.xls", sep = "\t", quote = F, row.names = F)
```

```
# Proportion table
df_ratio <- df_wide
num_cols <- setdiff(colnames(df_ratio), "secondcelltype")
df_ratio[num_cols] <- lapply(df_ratio[num_cols], function(x) round(prop.table(x), 4))
write.table(df_ratio, file = "group_ratio.xls", sep = "\t", quote = F, row.names = F)
```

Code Availability: Spatial Transcriptomic Analysis

```
## Clear workspace
rm(list = ls())

## Load libraries library(Seurat)
library(ggplot2)
library(patchwork)
library(data.table)
library(dplyr)
library(hdf5r)
library(viridis)

## Load SpaceRanger output (.h5 + spatial folder)
spatial_obj <- Load10X_Spatial(
data.dir = "SpaceRanger/",
filename = "Visium_spatial_obj_filtered_feature_bc_matrix.h5", slice = "spatial_obj"
)

spatial_obj@project.name <- "spatial_obj"
spatial_obj$orig.ident <- "spatial_obj"
spatial_obj # check structure

## QC plots
p1 <- VlnPlot(spatial_obj, features = "nCount_Spatial", pt.size = 0, cols = "red")
p2 <- SpatialFeaturePlot(spatial_obj, features = "nCount_Spatial") p1 | p2

## SCTransform normalization + PCA
spatial_obj <- SCTransform(spatial_obj, assay = "Spatial", verbose = FALSE)
spatial_obj <- RunPCA(spatial_obj, assay = "SCT", verbose = FALSE)

## PCA visualization
plot1 <- DimPlot(spatial_obj, reduction = "pca", group.by = "orig.ident")
plot2 <- ElbowPlot(spatial_obj, ndims = 20, reduction = "pca") plot1 + plot2

## Choose PCs and perform clustering pc.num <- 1:15
spatial_obj <- FindNeighbors(spatial_obj, reduction = "pca", dims = pc.num)
spatial_obj <- FindClusters(spatial_obj, verbose = FALSE)

## UMAP + spatial visualization
spatial_obj <- RunUMAP(spatial_obj, reduction = "pca", dims = pc.num)
```

```
p1 <- DimPlot(spatial_obj, reduction = "umap", label = TRUE)

p2 <- SpatialDimPlot(spatial_obj, label = TRUE, label.size = 3) p1 + p2

## Save the Seurat object
save(spatial_obj, file = "spatial_obj_st.Rds")

################################################################################
######
Parse spatial coordinates from tissue_positions_list.csv
position <- fread("SpaceRanger/spatial/tissue_positions_list.csv")
colnames(position) <- c("barcode", "in_tissue", "row", "col", "pxl_row", "pxl_col")
position <- position[position$in_tissue == 1, ] # keep only in- tissue spots

## Coordinate conversion
position$row <- as.numeric(position$row)
position$col <- as.numeric(position$col)
rownames(position) <- position$barcode

## Visualization: blue scatterplot of spatial position
ggplot(position, aes(x = row, y = col)) +
geom_point(color = "blue") + theme_bw()

## Match to Seurat metadata
meta <- spatial_obj@meta.data
position <- position[rownames(meta), ] # ensure same spot order
meta$x <- position$row
meta$y <- position$col

## Cluster overlay on spatial layout
p3 <- ggplot(meta, aes(x = x, y = y, color = seurat_clusters)) + geom_point(size = 1) +
theme_bw() +
labs(title = "Clusters on Spatial Coordinates") p2 + p3

## Save spatial positions and metadata
save(position, file = "spatial_obj_position.Rds")
save(spatial_obj, file = "spatial_obj_st.Rds")

embed_umap2 <- data.frame(
UMAP_1 = position$row,
UMAP_2 = position$col,
row.names = rownames(position)
```

```
)

spatial_obj[["space"]] <- CreateDimReducObject(
embeddings = as.matrix(embed_umap2),
key = "SPACE_",
assay = DefaultAssay(spatial_obj)
)

################################################################################
## Feature plots: expression visualization

# UMAP view
FeaturePlot(spatial_obj, features = "Prox1", pt.size = 0.3)

# Spatial view with viridis color
SpatialFeaturePlot(spatial_obj, features = "Prox1", pt.size = 2)

# Blended marker expression
FeaturePlot(
object = spatial_obj,
features = c("Prox1", "Ptprc"),
blend = TRUE,
blend.threshold = 0.5,
cols = c("lightgrey", '#B53E2B', '#476D87'),
pt.size = 0.3
```
